# Spatial patterns in soil physicochemical and microbiological properties in a grassland adjacent to a newly built lake

**DOI:** 10.1002/mbo3.912

**Published:** 2019-08-31

**Authors:** Jinsheng Li, Chan An, Jianying Shang, Tianchi Zhao, Qian Zhang, Xiaomeng Yang, Cheng Ren, Ding Huang, Kesi Liu, Xinqing Shao

**Affiliations:** ^1^ Department of Grassland Science, College of Animal Science and Technology China Agricultural University Beijing China; ^2^ Department of Soil and Water Science College of Resources and Environment Sciences China Agricultural University Beijing China; ^3^ National Field Station of Grassland Ecosystem at Guyuan County Zhangjiakou China

**Keywords:** bacteria, grazing grassland, soil property, soil water content, the newly built lake

## Abstract

Soil water content (SWC) is an important determinant for nutrient cycling and microorganism activity in the grassland ecosystem. Lakes have a positive effect on the water supply of the neighboring ecosystem. However, information evaluating whether newly built lakes improve the physiochemical properties and microorganism activity of adjacent grassland soil is rare. A 15‐hectare artificial lake with a 2 m depth was built on grazed grassland to determine whether the change of soil physiochemical properties and microorganism activity of the adjacent grassland depended on the distance from the lake. SWC and total nitrogen (TN) were greater within 150 m of the lake than at distances over 150 m from the lake. The total organic carbon (TOC) increased first at 100–150 m from the lake and then decreased. The soil microbial biomass and the bacterial and fungal contents increased with increasing years after the construction of the lake. Gram‐negative bacteria and methanotrophic bacteria were greater within a 30 m distance of the lake. Over 60 m away from the lake, Actinobacteria, gram‐positive bacteria, and anaerobic bacteria showed higher abundances. In the area near the lake (<250 m distance), microorganisms were strongly correlated with SWC, EC, TN, and TOC and greatly correlated with the changes of total phosphorous (TP) and pH when the distance from the lake was over 250 m. The results indicated that the newly built lake could be a driving factor for improving the physiochemical properties and microorganism activity of adjacent grassland soil within a certain range.

## Graphical abstract

The new‐built lake increased the soil water content, total nitrogen, and total organic carbon of near‐lake grassland. Soil microbial biomass, bacteria, and fungi content of adjacent grassland increased with the increase of the built‐time of lake. The new‐built lake changed the sensitivity and relationship between microorganisms and soil physiochemical properties of adjacent grassland.

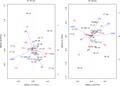

## 1 INTRODUCTION

The grassland ecosystems are the largest terrestrial ecosystem in the world and play an important role in global production and ecological services. In China, grasslands account for nearly 40% of the land area (Kang, Han, Zhang, & Sun, 2007), and most grasslands are in the arid and semiarid regions of northern China. In these regions, soil moisture is the main limiting factor for plant growth and an irreplaceable factor for maintaining the sustainable development of this region (Zhang, Shao, & Li, 2017). In the grassland ecosystem, soil moisture is an important determinant for nutrient cycling and flow in the system (Zou, Gao, & Fu, 2016). Relieving drought stress could accelerate the recovery of grasslands productivity (Hofer et al., 2016). Numerous studies have reported that changes in soil moisture over space and time have significant effects on soil nutrient elements and microbial activities (Hu et al., 2014; Martínez‐Murillo, Hueso‐González, & Ruiz‐Sinoga, 2017; McGovern et al., 2014; Teh, Rhew, & Atwood, 2010). There is a significant positive correlation between soil moisture and organic carbon (Tian et al., 2017). In grazing grasslands, Necpálová, Phelan, Casey, and Humphreys (2013) reported a positive correlation between soil available nitrogen and soil moisture content. Soil moisture also affects soil phosphorus migration and adsorption (Litaor, Reichmann, Haim, Auerswald, & Shenker, 2005).

In soil ecosystems, more than 95% of soil microorganisms live in the surface soil and play important roles in the cycle of nutrients, soil health and ecosystem productivity (Leeuwen et al., 2017; Moll et al., 2017; Pulleman, Six, Uyl, Marinissen, & Jongmans, 2005; Zelles, 1999). Soil microorganisms are sensitive to soil moisture changes, and changes in soil moisture directly affect the metabolism, quantity, and diversity of soil microorganisms (Brockett, Prescott, & Grayston, 2012; McMahon & Chapelle, 1991; Zhao et al., 2018). Changes in the structure and diversity of the soil microbial community were closely related to hydrological conditions (Foulquier, Volat, Neyra, Bornette, & Montuelle, 2013). In a tall‐grass prairie of the American Midwest, Fierer et al. (2013) found that the main driving factors of soil bacterial community diversity were the annual average precipitation and total precipitation. In a simulated rainfall experiment, Ma, Huang, Guo, Wang, and Xiao (2012) found that the carbon and nitrogen biomass and activity of soil microbes increased with increasing available water.

In grassland ecosystems, studies on the relationship among soil physiochemical properties, soil microorganisms, and moisture mostly focused on natural rainfall or simulated rainfall (Bastida et al., 2017; Guo, Li, et al., 2018; Guo, Weise, Fiedler, Lohmann, & Tietjen, 2018; Zhao et al., 2016), but the uncertainty and inhomogeneity of natural rainfall restricts its effectiveness for the evaluation of their dynamics and relationships. The presence of lakes generally has a positive effect on the water supply of neighboring ecosystems (Gong et al., 2017; Zhang, Zhao, & Zhang, 2012; Zhao et al., 2016). Therefore, if a lake were built on a grassland, then a simulated wet ecosystem with adjacent grasslands would be formed. The water shortage of grasslands in arid and semiarid regions could be mitigated. The soil environment, including soil physiochemical properties and soil microorganism activity, of adjacent grasslands would potentially improve. However, information related to the effect of the presence of a lake on the soil physiochemical traits and microbes of grasslands in the direct vicinity is rare. Therefore, this experiment studied the temporal and spatial responses of the physiochemical properties and microorganisms of grassland soil adjacent to a newly built lake. We hypothesized that (a) the soil water content of grasslands near the lake increased and the spatial distribution of soil physicochemical properties of adjacent grasslands changed after lake construction; and (b) the content of taxonomic groups of soil microbes increased with increasing years after the construction of the lake.

## 2 MATERIALS AND METHODS

### 2.1 Study site

The study was conducted in the National Field Station of Grassland Ecosystem at Guyuan County, Hebei Province, China (41°44′N, 115°42′E, 1,460 m a.s.l.). The entire station is located in a typical semiarid continental monsoon climate zone with an annual mean precipitation of 400 mm, approximately 80% of which falls in the growing season from July to September. The mean annual temperature is 1.4 ℃, and there are 80–95 frost free days. There are 2,930 annual hours of sunshine, and the annual evaporation is 1,735 mm. The soil in this region is classified as chestnut soil or Calciustepts by Chinese Soil Taxonomy and US Soil Taxonomy, respectively. The dominant plant in the study grassland was Chinese wildrye (*Leymus chinensis* Tzvel.), and the accompanying species were mainly alkaligrass (*Puccinellia distans*), *Potentilla acaulis*, *Suaeda glauca* (Bunge), and *Iris lactea*.

### 2.2 Experimental design and soil sampling

A 15‐hectare artificial lake with a 2 m depth was built on the grazing grassland in the National Field Station of Grassland Ecosystem at Guyuan County, Hebei Province, China, in June 2013 and completed in June 2014. The site of lake construction and the study grassland belonged to the same grazing grassland before the lake was constructed. The lake was constructed by digging the grazing grassland to a 2‐m depth and moving out the soil. Initial lake water mainly came from groundwater below 150 m. After water filling was completed, there was no dramatic fluctuation in the water table of the lake. To study the spatiotemporal responses of the soil physiochemical properties and microorganisms of the adjacent grassland to the newly built lake, eight 2 m wide × 2 m long × 2 m high iron cages made of fine iron bars were set perpendicularly at 10, 30, 60, 100, 150, 250, 400, and 600 m distances from the edge of the newly built lake in June 2014, which were named s1, s2, s3, s4, s5, s6, s7, and s8 in the text, respectively. The areas outside of the cages remained in their original grazing state (continuous grazing, 10 goats/ha), and the cages prevented the disturbance of animals from affecting the accuracy of data collection inside the cages.

Soil samples were collected from within cages during the peak growing period of plants (late July) in 2015, 2016, and 2017. Before we took soil samples, sunny days had generally occurred for more than one week. Three soil samples were randomly taken from the 0‐ to 10‐cm and 10‐ to 20‐cm soil layers at the given distance within one day. The collected soil samples from each layer and sampling site were divided into three parts. One part was placed into an aluminum box for soil water content (SWC) determination, one part was dried at 65°C for soil chemical analysis, and the third part was kept at −20°C for phospholipid fatty acid (PLFA) analysis.

#### 2.2.1 Soil physicochemical properties

Soil physiochemical properties included SWC, pH, electronic conductivity (EC), total organic carbon (TOC), total nitrogen (TN), and total phosphorus (TP). SWC was calculated as follows: SWC = (*W*_2_–*W*_3_)*100/(*W*_2_−*W*_1_), where *W*_1_ was the weight of the aluminum box, *W*_2_ was the weight of the initial soil sample with the aluminum box, and *W*_3_ was the weight of the dried soil sample with the aluminum box, which dried at 65°C to constant weight. Soil pH and electrical conductivity (EC) were measured with an acidity meter and conductivity meter (METTLER TOLEDO), respectively (Guo, Li, et al., 2018; Guo, Weise, et al., 2018). TOC was determined by the TOC determinator (MACRO) (Wang, Qin, Cai, Meng, & Zhang, 2014). TN was determined by the Kelvin determination apparatus (FOSS Kjeltec) (Ge, Li, Fan, Hou, & Liang, 2010). TP was measured by the sodium hydroxide fusion‐molybdenum antimony colorimetric method (JingHua) (Bao, 2013).

#### 2.2.2 Soil microorganisms

Phospholipid fatty acid (PLFA) analysis was used to measure the structure of the sedimentary microorganisms according to Bossio and Scow (Bossio & Scow, 1998). Briefly, 8‐g dry soil was extracted using a single‐phase mixture of chloroform/methanol/citrate buffer (23 ml at a 1:2:0.8 volume ratio). After phase separation, the CHCL_3_ layer (extracted lipids) was collected. The extract was transferred into a silica solid‐phase extraction column, and neutral lipids and glycolipids were removed by sequential elution with chloroform (5 ml) and acetone (10 ml). Phospholipids were then collected by elution with methanol (5 ml) and dried under N_2_. Afterward, the phospholipid fraction was methylated with a methanol–toluene solution and a potassium hydroxide methanol solution, and H_2_O (2 ml) and acetic acid (0.3 ml) were added. Fatty acid methyl esters were extracted in hexane (2 × 2 ml) and dried under N_2_. The resulting fatty acid methyl esters (FAME) were analyzed on an Agilent 6850 Gas Chromatograph (GC) with MIDI peak identification software. The column was an Agilent capillary column using H_2_ as the carrier gas. The GC temperature progression was controlled by the MIDI software. The fatty acid 19:0 was added as an internal standard before methylation. Identification and quantification of fatty acid methyl esters were conducted automatically by the MIDI peak identification software. The indicators of bacteria and fungi in PLFAs are shown in Table 1. The ratios of gram‐positive/gram‐negative bacteria and fungi/bacteria were analyzed.

**Table 1:** Microbial PLFA biomarkers and metrics

Community category	Community metric	PLFA biomarker	Reference
PLFA Biomass		Sum named	Tunlid et al. (1985)
Total Fungal PLFAs		16:1w5c, 18:1w9c 18:2w6,9c	
Total Bacterial PLFAs		15:0iso, 15:0anteiso 16:1w7c, 17:0 anteiso 17:0iso, 17:0cyclo 18:1w5c, 18:1w7c, 19:0 cyclo	Zelles, (1997); Zelles (1999)
F:B ratio		18:1w9c, 18:2w6, 9c/total bacterial	Bardgett, Hobbs, and Frostegård (1996); Kaur, Chaudhary, Kaur, Choudhary, and Kaushik (2005)
		PLFAs	
Indicator PLFAs	Gram‐positive bacteria	15:0iso	Kaur et al., (2005); Zelles (1997); Zelles, (1999)
	Anaerobic,	16:10 methyl	Ratledge and Wilkinson (1988)
	Gram‐negative bacteria	16:1w7c	Zelles, (1999; Ratledge and Wilkinson (1988)
	Arbuscular mycorrhizal fungi	16:1w5c	Olsson (1999)
	Saprotrophic fungi or Ectomycorrhizal fungi	18:1w9c	Bardgett et al. (1996); Frostegård, Tunlid, and Bååth (2011)
	Methanotrophic bacteria	18:1w7c	Sundh, Börjesson, and Tunlid (2000)
	Saprotrophic fungi	18:2w6,9c	Smith, Marínspiotta, and Balser (2015)
	Anaerobic, gram‐negative bacteria	19:0 cyclo	Vestal and White (1989)

### 2.3 Statistical analysis

The spatiotemporal responses of the soil physiochemical properties to the newly built lake were analyzed by analysis of variance (ANOVA) using the general linear model (GLM) procedure, with distance, years, and their interaction in the model. Least square means were used for mean separation. Multiple comparisons were used to compare the differences between distances for each year or among years within each distance. The spatiotemporal responses of soil microorganisms were analyzed using principal component analysis (PCA). The relationships between soil physiochemical properties and microorganisms were analyzed using detrended correspondence analysis (DCA) and redundant analysis (RDA). SWC, pH, EC, TOC, TN, and TP were used as environmental data, and the detected specific microorganisms were used as the species data. After the conversion of species and environmental data, all species data were first analyzed using DCA to select the suitable model for data analysis (Reavie, Hall, & Smol, 1995). To eliminate the arch effect, a single‐peak response value with a gradient length of <3 was achieved, which indicated that the species data had an obvious linear response relationship with the previous two environmental axes and were suitable for redundant analysis (RDA) (Jongman, Braak, & Tongeren, 1995). There was no abnormal species data in the RDA test, and the variance expansion factors (VIF) of environmental indicators were all lower than the normal value of 20; that is, the typical correlation coefficient of environmental indicators and the axis was stable, which was suitable for further explanation (Teixeira, Assis, Siqueira, & Casagrande, 2008). The relationship of microorganisms and soil physicochemical properties in different soil layers was analyzed using path analysis, and data from different years and distances were combined in path analysis. PCA, DCA, and RDA analysis was completed by the vegan package in R3.3.3, and the path analysis was completed by the sem package in R3.3.3. Effects were considered to be significant if *p* ≤ .05.

## 3 RESULTS

### 3.1 The responses of soil physiochemical parameters to the newly built lake

A newly built lake had a spatiotemporal effect on the SWC of adjacent grasslands (Table 2). In the 0‐ to 10‐cm soil layer, SWC decreased significantly as the distance from the lake increased from 10 to 150 m and decreased up to 19.02%, 15.48%, and 13.31% in 2015, 2016, and 2017, respectively. At more than 150 m from the lake, SWC was not different among distances. At most of the distances, except for at 10 and 150 m, SWC had no much difference among years since lake construction. In the 10‐ to 20‐cm soil layer, SWC within a 100 m distance decreased significantly with increasing distance from the lake, falling by 10.96%, 10.94%, and 4.38%, in 2015, 2016, and 2017, respectively. SWC did not change significantly among distances >100 m from the lake. There was no significant interannual difference at most of the distances except 10 and 400 m.

**Table 2:** Changes in soil water content (SWC), electrical conductivity (EC), and pH within different soil layers at different distances from lake during 2015, 2016, and 2017

Parameter	Year	Layer (cm)	Distance from lake (m)							
			10	30	60	100	150	250	400	600
SWC (%)	2015	0–10	46 ± 1.4aA	39 ± 5.4abA	33 ± 8.2bcA	32 ± 4.5bcA	27 ± 1.8cdAB	20 ± 2.1dA	18 ± 1.1dA	21 ± 1.5cdA
	2016		33 ± 0.7aC	36 ± 0.3aA	29 ± 0.1bA	25 ± 4.5cA	21 ± 2.0dB	18 ± 1.1dA	19 ± 0.0dA	21 ± 0.6dA
	2017		41 ± 0.0aB	41 ± 4.6aA	38 ± 1.5abA	28 ± 5.0cA	31 ± 2.3bcA	20 ± 0.7cA	20 ± 0.2cA	19 ± 3.2cA
	2015	10–20	31 ± 0.9aA	32 ± 3.9aA	25 ± 1.8bA	21 ± 1.2cA	20 ± 4.7dA	20 ± 1.6dA	19 ± 0.6dA	17 ± 1.4d
	2016		30 ± 0.8aAB	28 ± 0.9aA	23 ± 1.9abA	19 ± 2.5bcA	17 ± 0.3bcA	16 ± 0.6bcA	17 ± 0.6bcB	14 ± 0.2c
	2017		26 ± 1.1aA	29 ± 1.1aA	26 ± 0.1aA	22 ± 2.2abA	23 ± 4.0abA	16 ± 1.6bA	12 ± 1.0Cc	11 ± 0.1b
EC (μs/cm)	2015	0–10	997 ± 63.7bA	1,711 ± 191abA	1,632 ± 160.9abA	2,840 ± 43.6abA	4,816 ± 15.3aA	1,201 ± 47.3bA	1,476 ± 98.6abA	830 ± 10.6bB
	2016		1,166 ± 263.2abA	1,191 ± 89.5abB	1,187 ± 91.8abB	1,536 ± 240.8aB	1,182 ± 143.1abB	766 ± 207.4bB	805 ± 159.5bB	1,592 ± 239.3aA
	2017		277 ± 80.4cB	690 ± 221.3cC	1,180 ± 96.1bcB	1,432 ± 226.1aB	1,435 ± 223.8aB	532 ± 156.9cB	776 ± 79.2bB	372 ± 23.3cC
	2015	10–20	751 ± 6.2dA	860 + 50.7dB	1,444 ± 79.2bA	2,397 ± 167.7aA	2,447 ± 274.7aA	874 ± 225.2cdA	132 ± 115.5bcA	1,285 ± 9.9bcA
	2016		947 ± 70.2dA	961 ± 26.0dA	1,677 ± 71.1aA	1,519 ± 302.2abB	1,061 ± 281.4bcB	853 ± 192.6dA	1,464 ± 56.4bcA	1,001 ± 67.7cdB
	2017		664 ± 503.8cA	338 ± 11.9cC	957 ± 290.3bB	1,640 ± 101.7aB	1,476 ± 42.1abB	790 ± 11.3bA	1,009 ± 57.3aB	41 ± 40.5cC
pH	2015	0–10	8.58 ± 0.09abA	8.60 ± 0.03abA	8.38 ± 0.06abA	8.22 ± 0.05bcA	7.96 ± 0.08cA	8.67 ± 0.07aA	8.47 ± 0.32abA	8.73 ± 0.136aA
	2016		10.09 ± 0.05bA	10.49 ± 0.09aA	9.54 ± 0.16cA	9.46 ± 0.11cdA	9.44 ± 0.10cA	9.18 ± 0.11dA	9.17 ± 0.09dA	9.43 ± 0.09cA
	2017		8.90 ± 0.30bA	9.47 ± 0.08aA	9.96 ± 0.03aA	8.51 ± 0.28bA	8.61 ± 0.30bA	8.22 ± 0.03cA	8.12 ± 0.02cA	7.92 ± 0.20cA
	2015	10–20	8.67 ± 0.04cB	8.91 ± 0.04aC	8.86 ± 0.06abC	8.43 ± 0.06dC	8.33 ± 0.04dC	8.89 ± 0.03aC	8.76 ± 0.03cB	8.76 ± 0.5cdB
	2016		9.44 ± 0.09cA	10.64 ± 0.06aA	9.42 ± 0.04dB	9.57 ± 0.17bcA	9.92 ± 0.26bA	9.23 ± 0.05dB	9.79 ± 0.07bA	9.82 ± 0.18bA
	2017		9.22b ± 0.45AB	9.25 ± 0.03bB	9.94 ± 0.01aA	9.09 ± 0.26bB	9.20 ± 0.07bB	9.49 ± 0.6abA	8.75 ± 0.01cB	8.95 ± 0.01bcB

Note

Data are means ± *SD*; means in rows within each year with different lowercase letters are significantly different at *p ≤ *.05; means in columns within each distance from lake with different uppercase letters are significantly different at *p ≤ *.05.

The surface soil EC had a fluctuating change with the distance from the lake and the years since construction of lake (Table 2). In the 0‐ to 10‐cm soil layer, EC first increased and then decreased as the distance from the lake increased. The maximum values were 4,816 μs/cm at 150 m in 2015, 1,536 μs/cm at 100 m in 2016, and 1,435 μs/cm at 150 m in 2017. In the 10‐ to 20‐cm soil layer, the soil EC showed a similar trend as that of the 0‐ to 10‐cm soil layer, which first increased and then decreased with increasing distance from the lake. The maximum values were 2,447 μs/cm at 150 m in 2015, 1,677 μs/cm at 60 m in 2016, and 1,640 μs/cm at 100 m in 2017. As the years since the construction of the lake increased from 2015 to 2017, the soil EC in the 0‐ to 20‐cm soil layer decreased.

The soil pH showed different fluctuations in different years with increasing distance from the lake (Table 2). In the 0‐ to 10‐cm soil layer, the pH in 2015 first decreased until 150 m and then increased with increasing distance from the lake, reaching a minimum value of 7.96 at 150 m. In 2016, the pH was significantly higher in the area near the lake (10–30 m) than in the area farther from the lake (60–600 m). Within the distance of 150 m, pH values in 2017 were greater than in 2015; however, pH values within 250–600 m of the lake were smaller than those in 2015. In the 10‐ to 20‐cm soil layer, the pH value at a given distance increased significantly with increasing years since the construction of the lake; that is, pH values from 2016 and 2017 were significantly greater than those from 2015.

The newly built lake affected the spatial and temporal distribution of soil TN, TP, and TOC in adjacent grasslands (Table 3). In 2015, in the 0‐ to 10‐cm soil layer, the soil TN first increased until 150m and then decreased with increasing distance from the lake, reaching a maximum of 3.13 g/kg at 150 m (Table 3). In 2016, soil TN within 150 m had no significant change, but the value gradually decreased when the distance from the lake exceeded 150 m. In 2017, soil TN first increased until 30 m and then decreased. The soil TP was generally greater in the area near the lake (within 150 m) in 2015, 2016, and 2017 than in the area farther from the lake. The soil TOC in 2015 first increased until 150m and then decreased with increasing distance from the lake, reaching a maximum of 26.87 g/kg at 150 m (Table 3). In 2016 and 2017, soil organic carbon within 150 m of the lake was generally greater than that at more than 150 m distance.

**Table 3:** Changes in soil total nitrogen (TN), total phosphorous (TP), and total organic carbon (TOC) within different soil layers at different distances from the lake during 2015, 2016, and 2017

Parameter	Year	Layer (cm)	Distance from lake (m)							
			10	30	60	100	150	250	400	600
TN (g/kg)	2015	0–10	2.48 ± 0.14dB	2.96 ± 0.01bcA	2.97 ± 0.09bA	3.00 ± 0.02abA	3.13 ± 0.05aA	2.84 ± 0.05cA	2.49 ± 0.31dA	2.50 ± 0.4dA
	2016		2.99 ± 0.14aA	2.76 ± 0.02bA	3.00 ± 0.02aA	2.82 ± 0.05bA	2.78 ± 0.05bB	2.60 ± 0.07cA	2.56 ± 0.10cA	2.16 ± 0.06dB
	2017		2.19 ± 0.16bB	3.78 ± 0.80aA	2.92 ± 0.06abA	2.82 ± 0.11abA	2.88 ± 0.05abB	2.74 ± 0.18abA	2.42 ± 0.01bA	2.36 ± 0.06bA
	2015	10–20	1.58 ± 0.06dA	1.69 ± 0.05cdA	1.75 ± 0.05cdB	1.84 ± 0.02bcA	2.67 ± 0.01aA	1.66 ± 0.06cdA	1.91 ± 0.13bA	1.46 ± 0.02dA
	2016		1.60 ± 0.07dA	1.73 ± 0.01cA	1.87 ± 0.07bB	1.69 ± 0.05cB	2.26 ± 0.07aA	1.46 ± 0.063eA	1.75 ± 0.01cAB	1.29 ± 0.03eA
	2017		1.59 ± 0.15bA	1.48 ± 0.13bA	2.39 ± 0.07aA	1.92 ± 0.01abA	2.22 ± 0.27aA	1.44 ± 0.27bA	1.50 ± 0.07bB	1.45 ± 0.3bA
TP (g/kg)	2015	0–10	0.19 ± 0.05bcA	0.25 ± 0.03abAB	0.22 ± 0.01abA	0.10 ± 0.01dAB	0.09 ± 0.04dB	0.15 ± 0.02cdA	0.29 ± 0.02aA	0.16 ± 0.01cdA
	2016		0.24 ± 0.04abA	0.29 ± 0.02aA	0.19 ± 0.01bcA	0.25 ± 0.08abA	0.19 ± 0.01bcA	0.13 ± 0.01dA	0.25 ± 0.02aA	0.14 ± 0.02cdA
	2017		0.19 ± 0.02aA	0.17 ± 0.01aB	0.19 ± 0.03aA	0.20 ± 0.01aB	0.19 ± 0.01aA	0.14 ± 0.02bA	0.19 ± 0.03aA	0.14 ± 0.02bA
	2015	10–20	0.14 ± 0.02abA	0.10 ± 0.4cA	0.17 ± 0.01aA	0.09 ± 0.02bA	0.15 ± 0.02abA	0.21 ± 0.05aA	0.19 ± 0.03aA	0.08 ± 0.001bA
	2016		0.19 ± 0.05abA	0.22 ± 0.02aA	0.15 ± 0.02bcA	0.10 ± 0.02cA	0.16 ± 0.04bcA	0.14 ± 0.03bcA	0.12 ± 0.02cA	0.13 ± 0.02bcA
	2017		0.16 ± 0.004aA	0.15 ± 0.004aA	0.12 ± 0.04abA	0.15 ± 0.003aA	0.16 ± 0.01aA	0.11 ± 0.03abA	0.14 ± 0.02aA	0.07 ± 0.004bA
TOC (g/kg)	2015	0–10	12.34 ± 1.46cB	17.39 ± 2.38bcA	24.00 ± 7.19abA	22.55 ± 1.53abAB	26.87 ± 4.68aA	18.99 ± 3.53bcA	19.03 ± 1.05bcA	16.25 ± 2.63cdA
	2016		26.52 ± 3.25aA	20.35 ± 3.26bcA	18.44 ± 2.25bcA	19.37 ± 1.52bcB	21.24 ± 5.71abA	18.08 ± 1.03bcA	11.83 ± 3.69cA	16.41 ± 3.49bcA
	2017		10.43 ± 0.16abB	3.32 ± 1.55cB	23.97 ± 5.99aA	25.10 ± 2.57aA	23.11 ± 2.71abA	20.22 ± 2.20abA	10.45 ± 2.25bA	19.45 ± 1.94abA
	2015	10–20	7.84 ± 1.22bA	9.10 ± 1.09bA	11.74 ± 0.89bAB	13.52 ± 1.03bA	21.76 ± 7.61aA	8.71 ± 0.04bA	12.18 ± 3.34bA	9.66 ± 1.75bB
	2016		8.55 ± 2.59bcA	12.83 ± 2.88abA	16.56 ± 3.42aA	11.66 ± 0.28abA	16.62 ± 1.43aA	10.26 ± 0.30bA	10.03 ± 0.74bA	6.63 ± 1.24cB
	2017		1.09 ± 0.01bB	1.52 ± 1.24bB	7.83 ± 1.09abB	10.09 ± 3.17abA	16.47 ± 1.48aA	14.31 ± 4.14aA	13.53 ± 3.02aA	13.67 ± 0.77aA

Note

Data are means ± *SD*; means in rows within each year with different lowercase letters are significantly different at *p* ≤ .05; means in columns within each distance from lake with different uppercase letters are significantly different at *p* ≤ .05.

In the 10‐ to 20‐cm soil layer, soil TN showed a similar trend in 2015, 2016, and 2017, increasing until 150 m and then decreasing with the change of distance (Table 3). The soil TP decreased with increasing distance from the lake each year, but the differences among distances decreased with increasing lake establishment time. The soil TOC first increased and then decreased with increasing distance from the lake, reaching a maximum at 150 m. When the years since the construction of the lake increased from 2015 to 2017, the soil TOC decreased within 60 m distance and increased at 600 m distance; however, there was no difference in the distance from 100 to 400 m.

### 3.2 Responses of soil microorganisms to the newly built lake

**Figure 1:**
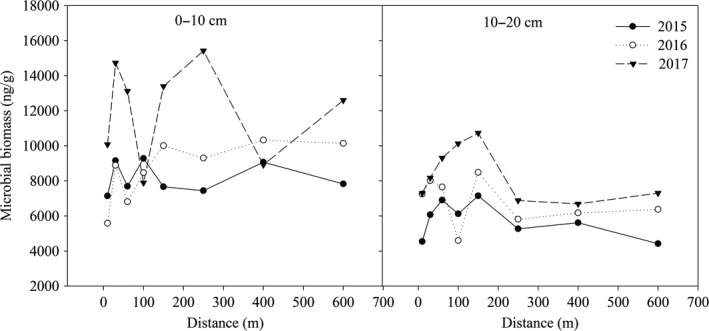
The variation of soil microbial mass within different soil layers at different distances from lake during 2015, 2016, and 2017

The soil microbial biomass (SMB) changed dynamically in different soil layers and at different distances from the lake (Figure 1). In the 0‐ to 10‐cm soil layer, SMB within 10–150 m from the lake fluctuated greatly in 2015 and 2016; however, the fluctuation of SMB became lower with a distance from the lake of more than 150 m. In 2017, SMB within 300 m of the lake was generally greater than that farther from the lake (>300 m). With the increase in years since the construction of the lake, the SMB at most distances (except for 100 and 400 m) in 2017 was greater than that in 2015 and 2016. In the 10‐ to 20‐cm soil layer, SMB at most distances (except for 100 m) increased with increasing years since the construction of the lake, and the highest SMB values in the past three years occurred at 150 m, which were 7,148 ng/g in 2015, 8,485 ng/g in 2016, and 10,735 ng/g in 2017. The fluctuation of SMB among distances became lower when the distance was over 150 m from the lake.

**Figure 2:**
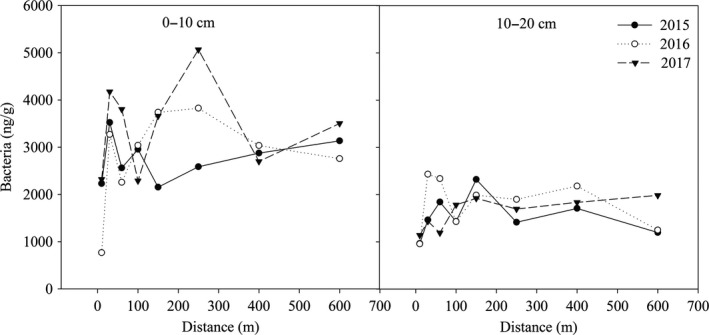
The variation of soil bacterial content within different soil layers at different distances from the lake during 2015, 2016, and 2017

The bacterial content in the 0‐ to 10‐cm soil layer fluctuated greatly with the change of distance from the lake and was especially high within 150 m of the lake in 2015 and 2016 (Figure 2). When the distance was over 150 m from the lake, the bacterial content increased in 2015 and decreased in 2016 with increasing distance. With the increase in years since the construction of the lake, the bacterial content increased at most distances (except 100 and 400 m). In the 10‐ to 20‐cm soil layer, the bacterial content at 10 m was the lowest. In 2015, the bacterial content first increased and then decreased with increasing distance from the lake, and the maximum value was 2,318.35 ng/g at 150 m. The change in bacterial content in 2016 was similar to that in 2015, but the magnitude of the change decreased when the distance was over 100 m from the lake. In 2017, the bacterial content within 100 m of the lake was less than that from 100 to 600 m, and the bacterial content from 100 to 600 m was almost unchanged, ranging from 1,692.88 to 1,981.83 ng/g.

**Figure 3:**
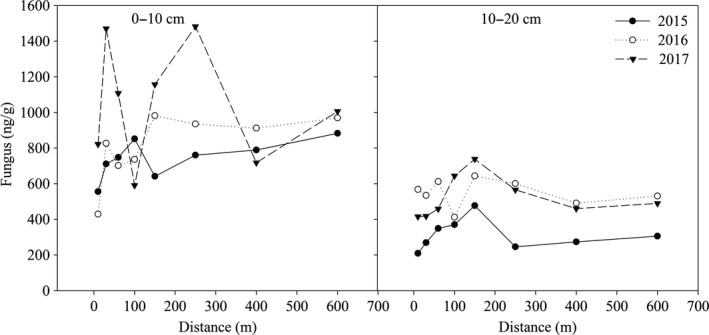
The variation of soil fungal content within different soil layers at different distances from the lake during 2015, 2016, and 2017

The fungal content in the 0‐ to 10‐cm soil layer within 150 m of the lake in 2015 first increased and then decreased (Figure 3). The minimum and maximum values were 555.38 ng/g at 10 m and 852.06 ng/g at 100 m. When the distance was more than 150 m, the fungal content increased slightly with increasing distance. In 2016, the fungal content within 10–150 m of the lake generally increased with increasing distance, reaching a maximum of 982.11 ng/g at 150 m. When the distance was more than 150 m, the fungal content fluctuated slightly among distances, ranging from 911.79 to 969.91 ng/g. In 2017, the fungal content fluctuated greatly among distances from the lake, ranging from 591.07 ng/g at 100 m to 1,482.62 ng/g at 250 m. When the years since the construction of the lake increased from 2015 to 2017, the fungal content increased across all distances. In the 10‐ to 20‐cm soil layer, the fungal content generally increased first and then decreased as the distance increased in each year. Compared with 2015, the fungal content at all distances showed an increasing trend with increasing years since the construction of the lake.

**Figure 4:**
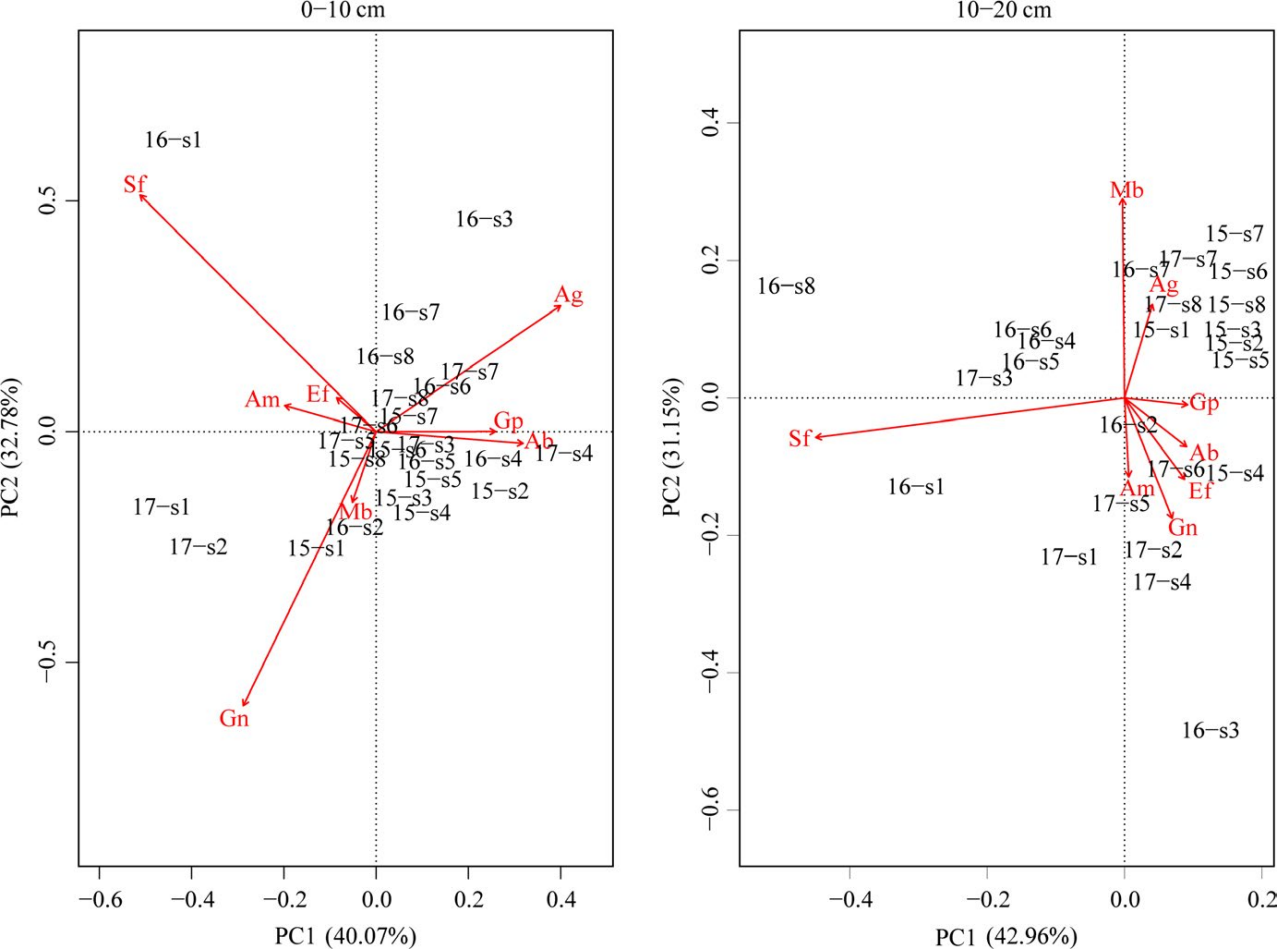
Principal component analysis of soil microbial community within different soil layers at different distances from the lake during the years 2015, 2016, and 2017. Gp (gram‐positive bacteria), Ab (Actinobacteria), Gn (gram‐negative bacteria), Am (arbuscular mycorrhizal fungi), Ef (ectomycorrhizal fungi), Mb (methanotrophic bacteria), Ag (anaerobic bacteria), and Sf (saprotrophic fungi). 15‐s1 to 15‐s8 represents distance in 2015; 16‐s1 to 16‐s8 represents distance in 2016; 17‐s1 to 17‐s8 represents distance in 2017

In the 0‐ to 10‐cm soil layer, the first and second sequence axes of the PCA explained 40.07% and 32.78% of the variation in microbial activity, respectively (Figure 4). In the microbial community, saprotrophic fungi, gram‐negative bacteria, and anaerobic bacteria had the largest contribution to the first and second sequence axes. In 2015, the first year after the lake was built, gram‐positive bacteria and Actinobacteria showed a greater content at most distances compared to saprotrophic fungi, arbuscular mycorrhizal fungi, and ectomycorrhizal fungi. Gram‐negative bacteria and methanotrophic bacteria had relatively high contents at 10 and 600 m from the lake compared to other distances. In 2016, saprotrophic fungi, arbuscular mycorrhizal fungi, and ectomycorrhizal fungi were greater at a 10 m distance. Gram‐negative bacteria and methanotrophic bacteria were high at 30 m. At a distance of 60–600 m, the anaerobic bacteria, gram‐positive bacteria, and Actinobacteria contents were high. In 2017, gram‐negative bacteria and methanotrophic bacteria were greater within 10–30 m distance, Actinobacteria, gram‐positive bacteria, and anaerobic bacteria had high contents when the distance was more than 60 m from the lake. In the 10‐ to 20‐cm soil layer, the first and second sequence axes explained 42.96% and 31.15% of the variation in microbial activity, respectively (Figure 4). The values of distance in 2015 were mostly distributed in the first quadrant, and methanotrophic and anaerobic bacteria contents were high. In 2016, soil saprotrophic fungi content increased, and soil arbuscular mycorrhizal fungi, gram‐negative bacteria, and ectomycorrhizal fungi contents at 10 and 30 m from the lake increased as well. In 2017, arbuscular mycorrhizal fungi, gram‐negative bacteria, ectomycorrhizal fungi, and saprotrophic fungi had higher contents within a 250 m distance. Methanotrophic bacteria and anaerobic bacteria contents were higher when the distance from the lake was 400 and 600 m, respectively.

### 3.3 Relationship between microorganisms and soil physiochemical parameters

**Figure 5:**
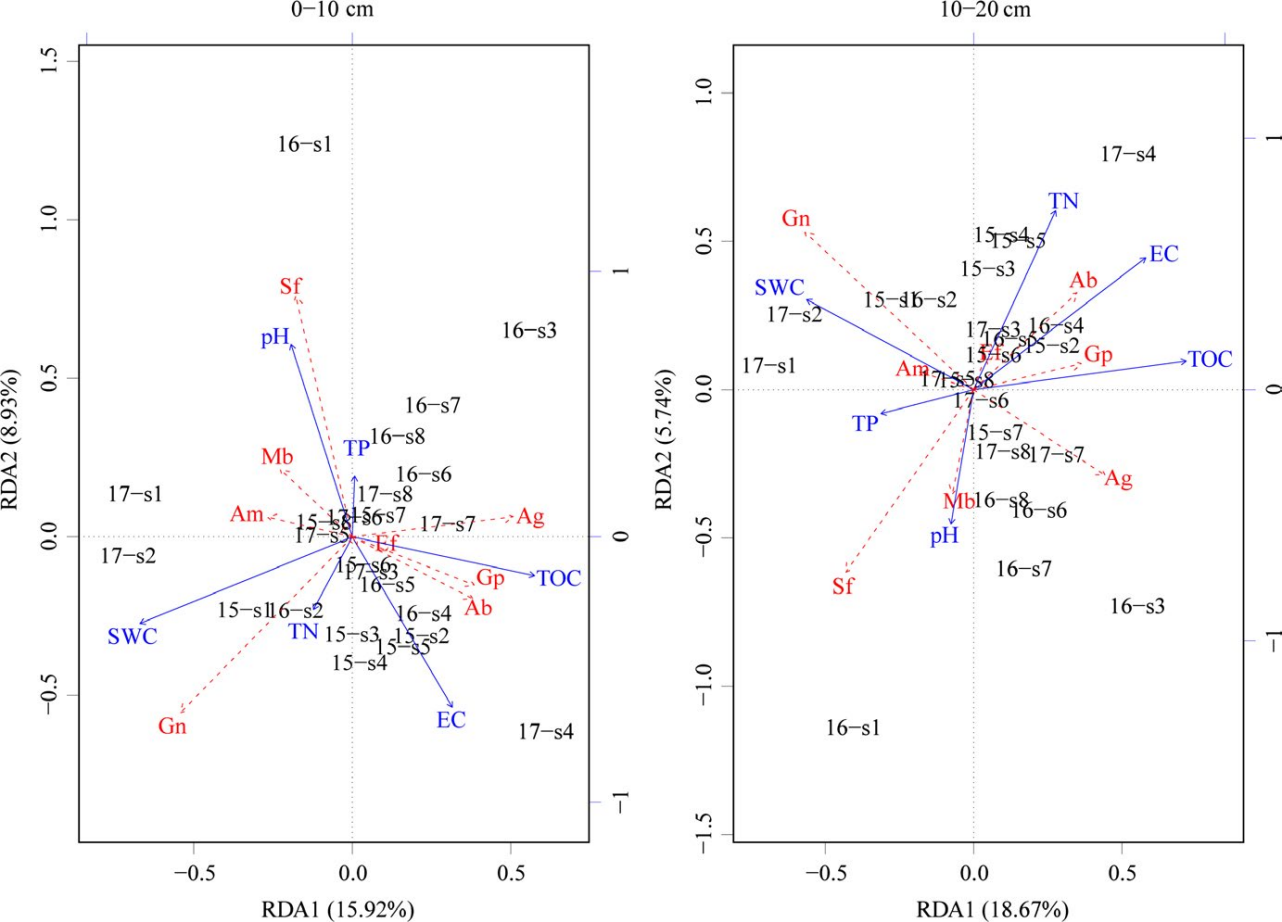
RDA two‐dimensional sequencing diagram of microbial and soil physicochemical properties in different soil layers. The blue lines represent different soil physicochemical indexes, and the red dashed lines represent different microorganism indicators. SWC represents soil water content, EC represents electrical conductivity, TOC represents soil organic carbon, TP represents soil total phosphorus, and TN represents soil total nitrogen

In the 0‐ to 10‐cm soil layer, microorganisms had varied correlations with different soil physiochemical parameters (Figure 5). SWC and pH contributed most to the variation in microbial communities. SWC was positively correlated with gram‐negative bacteria and negatively correlated with gram‐positive bacteria. pH was positively correlated with saprotrophic fungi and negatively correlated with Actinobacteria. In 2015, the soil microorganisms at 10–400 m distances from the lake were sensitive to changes in SWC, TN, and EC. The soil microorganisms were sensitive to changes in pH and TP when the distance from the lake was 400 m. In 2016, the values of microorganisms at each distance showed a more dispersed distribution in the RDA graph than in 2015, and the soil microorganisms within a distance of 250–600 m were more sensitive to changes in pH and TP. In 2017, the soil microorganisms were sensitive to the changes of SWC, EC, and TOC, respectively, when the soil microorganisms were at the 10–30 m, the 60–100 m, and the 150–600 m distances, respectively. In the 10‐ to 20‐cm soil layer, TN, EC, TOC, and SWC showed the major contributions to microbial activity, as shown on the RDA map. TN, EC, and TOC were positively correlated with Actinobacteria and gram‐positive bacteria and negatively correlated with saprotrophic fungi and arbuscular mycorrhizal fungi. SWC had a positive correlation with gram‐negative bacteria and a negative correlation with anaerobic bacteria. In 2015, the soil microorganisms at the 10–250 m distance were sensitive to changes in TN, EC, TOC, and SWC. In 2016, the soil microorganisms at a distance of 10, 60, and 250–600 m were sensitive to changes in TP and pH. The soil microorganisms at other distances were sensitive to changes in TN and EC. In 2017, the values of microorganisms at each distance showed a more dispersed distribution in the RDA map than in 2015 and 2016. The soil microorganisms were sensitive to the changes in SWC at the 10–30 m distance, to changes in TN and EC at the 60–150 m distance, and to changes in TP and pH at the 250–600 m distance, respectively.

**Figure 6:**
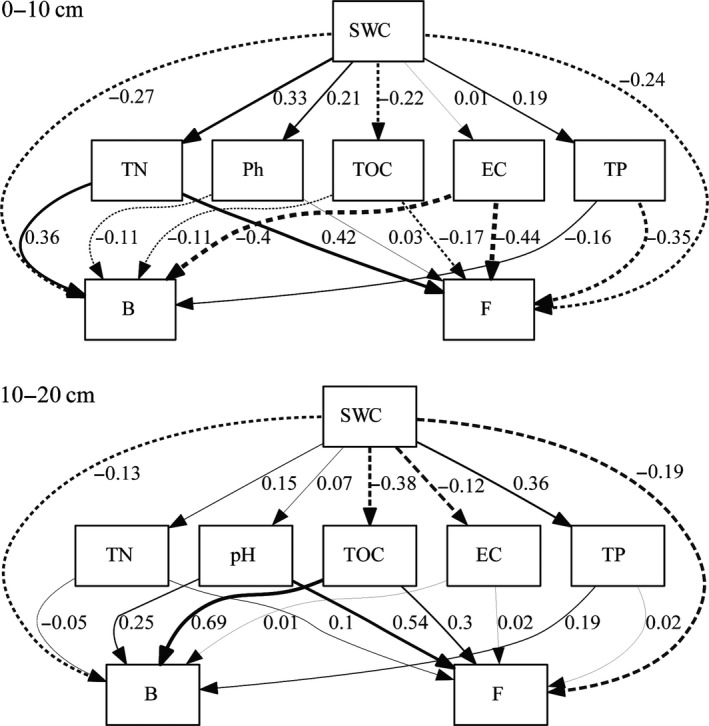
The path analysis of the relationship of microorganisms and soil physicochemical properties in different soil layers. B represents bacteria, F represents fungi, the dashed line represents a negative correlation, the solid represents a positive correlation, and the thickness of the line represents the strength of the correlation

In the 0‐ to 10‐cm soil layer, SWC was negatively correlated with bacteria and fungi, and the path coefficients were −0.27 and −0.24, respectively (Figure 6). The effect of SWC on soil chemical properties was mostly positive, except for a negative correlation with TOC. Bacteria had a positive correlation with TN and a negative correlation with pH, TOC, EC, and TP. Among these, TN and EC had a great influence on bacterial contents, and the path coefficients were 0.36 and −0.40, respectively. Fungal content was positively correlated with TN and pH and negatively correlated with TOC, EC, and TP. Among these, TN, EC, and TP had relatively great effects on fungal content, with path coefficients of 0.42, −0.44, and −0.35, respectively. In the 10‐ to 20‐cm soil layer, SWC had a negative effect on bacteria and fungi, and the path coefficients were −0.13 and −0.19, respectively. Between SWC and other soil properties, SWC was positively correlated with TN, pH, and TP and negatively correlated with TOC and EC; the path coefficients were 0.15, 0.07, 0.36, −0.38, and −0.12, respectively. Bacterial content was positively correlated with pH, TOC, EC, and TP and negatively correlated with TN. Among these, TOC had relatively great effects on bacterial contents, with a path coefficient of 0.69. Fungi content had a positive correlation with TN, pH, TOC, EC, and TP. Among these, pH had relatively great effect on fungal content, with a path coefficient of 0.54.

## 4 DISCUSSION

### 4.1 Response of soil physiochemical properties to the newly built lake

When the new lake was built on grassland in this study, it changed the soil environment of the adjacent grassland to some extent. The spatial variation of soil physicochemical properties of the grassland occurred following the appearance of the lake. The SWC decreased gradually with increasing distance from the lake and finally became stable. The SWC of the 0‐ to 10‐cm soil layer became stable when the distance was more than 150 meters, while the SWC of 10‐ to 20‐cm soil layer tended to be stable when the distance was more than 100 m. At the same distance, the magnitude of change in the SWC in the 0‐ to 10‐cm soil layer was greater than that in the 10‐ to 20‐cm soil layer. These results indicated that the influence of the newly built lake on SWC of the adjacent grassland had a certain range and was greater in the upper layer than in the lower layer of soil. These results verified the findings of Qiu, Fu, Wang, and Chen (2001), who found that environmental influences on soil became weak when soil depth increased.

In the 0‐ to 10‐cm soil layer, the pH value close to the lake (10–60 m distance) was greater than that at more than 100 m distance. This was probably because the SWC close to the artificial lake was higher. The higher SWC caused the increase in ${\text{MnO}}_{2}^{+}$, FeOH^+^, and ${\text{SO}}_{4}^{2-}$ contents in soil and the decrease in H^+^ contents due to consumption and thus increased the soil pH value (Willett, 1989). However, this phenomenon in deep soil (10–20 cm) was not apparent, possibly because secretions from roots in this soil layer balanced the change in pH (Sarabia et al., 2018). The EC of different soil layers first increased and then decreased with increasing distance from the lake, reaching a maximum within 100–150 m distance. This change was probably associated with the change of SWC. Carsel and Parrish (1988) reported that water has a great influence on soil salinity dynamics. In this study, the high SWC at 10–60 m distance could dissolve the salinity brought by evaporation, while the low SWC at 250–600 m could not bring salt to the upper soil through evaporation.

Environmental changes also affected the spatial distribution of soil nutrients. In this study, TN, TP, and TOC in the surface soil (0–10 cm) were higher than that in the deep soil (10–20 cm). This was probably because the surface soil had good air permeability, a large amount of litter and high microbial activity, which promoted the accumulation of soil nutrients (Ma et al., 2015). However, under extreme water conditions (drought or flooding), the decreased microbial activity affected decomposition and then decreased soil nutrient contents (Kucera & Kirkham, 1971). In this study, soil TN and TOC first increased and then decreased with increasing distance from the lake, which was probably because the high SWC in the area near the lake affected the microbial activity and then resulted in relatively low nutrient contents in the same area. The TP of different soil layers at 10 and 30 m was significantly higher than that at other distances farther from the lake. This is probably because the high water content in the area near the lake led to the death of less tolerant microorganisms with a large amount of fatty acids and thus increased the soil phosphorus content (Blackwell et al., 2009).

### 4.2 Response of soil microorganisms to the newly built lake

Soil organisms play an important role in the decomposition of soil organic matter and mineralization of nutrient elements, but they are very sensitive to the soil environment. Changes in the environment can lead to changes in microbial community structure (Li et al., 2015). In this study, microbial biomass and bacterial and fungal contents at most distances decreased with increasing soil depth. This may be because the upper soil (0–10 cm soil depth) has better exchange with external conditions such as water, gas, and nutrients, which provide better conditions for the growth and metabolism of microorganisms (Stone, Deforest, & Plante, 2014). In addition, the topsoil contained the most resources (higher TOC and TN contents) that were generally strong drivers of microbial biomass. Therefore, microorganisms in the upper soil were more active than those in the lower soil. In the 10‐ to 20‐cm soil layer, the fluctuation of the biomass of microorganisms, bacteria, and fungi was greater within 250 m distance from lake than at farther distances. This was probably because the impact of the lake on the surrounding environment had a certain range, so it showed low magnitude when the distance from the lake exceeded a certain range. In 2017, the third year after the lake was built, the amounts of microorganisms, bacteria, and fungi at most distances in the surface soil were greater than those in 2015 and 2016. The improved soil water conditions could facilitate the decomposition of litter (Smith, Woodin, Pakeman, Johnson, & Van, 2014). Decomposition of litter increases the release of nutrients (Austin & Ballaré, 2010), and more nutrients accelerate the growth of microorganisms (Zönnchen, Schaaf, & Esperschütz, 2014).

Among the main soil physical and chemical factors, SWC and pH had the greatest impact on soil microorganisms in the 0‐ to 10‐cm soil layer or the 10‐ to 20‐cm soil layer. Soil water content is an important driving factor influencing soil microbial processes (Foulquier et al., 2013). Under water pressure, a series of small molecules will be produced in microbial cells to adapt to the new water environment (Schimel, Scott, & Killham, 1989). Due to the different structures of microorganisms, water conditions of microorganisms needed for survival are different, which leads to different microorganism community structures under varied water contents (Harris, 1981; Wood et al., 2001). Our results verified the relationship between SWC and microorganisms. SWC was positively correlated with gram‐negative bacteria and negatively correlated with gram‐positive bacteria. Many studies have also noted that soil pH value is an important driver of microbial community structural changes (Abbasi & Adams, 2000; Hassink, 1992). This is because most of the reactions in the growth processes of microorganisms are enzymatic reactions. Enzymatic reactions need to be carried out within a certain pH range.

## 5 CONCLUSION

Grassland in arid and semiarid region generally faced the problem of water shortage. Constructing a lake on the grassland could mitigate this issue to some degree, especially for grassland area close to the lake. After the lake was constructed, SWC, TN, and TOC generally had greater values within 150 m of the lake. The spatial distributions of microorganisms of adjacent grassland soil were also affected after the construction of the lake. The SMB and the bacterial and fungal contents generally increased with increasing years since the construction of the lake. Gram‐negative bacteria and methanotrophic bacteria were greater within a 30 m distance of the lake. Actinobacteria, gram‐positive bacteria, and anaerobic bacteria showed higher abundances over 60 m away from the lake. The results indicated that the newly built lake could be a driving factor for improving the physiochemical properties and microorganism activity of adjacent grassland soil within a certain range.

## CONFLICT OF INTERESTS

The authors declare no conflict of interest.

## AUTHOR CONTRIBUTIONS

Kesi Liu and Xinqing Shao performed the conceptualization and involved in the funding acquisition. Jingsheng Li and Kesi Liu were involved in the formal analysis. Chan An, Tianchi Zhao, Qian Zhang, Xiaomeng Yang, and Cheng Ren investigated the study report. Jinsheng Li and Kesi Liu wrote and drafted the original manuscript. Jianying Shang, Ding Huang, Kesi Liu, and Xinqing Shao reviewed and edited the manuscript.

## ETHICS STATEMENT

None required.

## DATA AVAILABILITY STATEMENT

All data generated or analyzed during this study are included in this published article.
